# Boosting working memory: uncovering the differential effects of tDCS and tACS

**DOI:** 10.1093/texcom/tgac018

**Published:** 2022-05-07

**Authors:** Daniel Senkowski, Rabea Sobirey, David Haslacher, Surjo R Soekadar

**Affiliations:** Charité - Universitätsmedizin Berlin, Freie Universität Berlin and Humboldt-Universität zu Berlin, Department of Psychiatry and Neurosciences, Charité Campus Mitte (CCM), Charitéplatz 1, 10117 Berlin, Germany; Charité - Universitätsmedizin Berlin, Freie Universität Berlin and Humboldt-Universität zu Berlin, Department of Psychiatry and Neurosciences, Charité Campus Mitte (CCM), Charitéplatz 1, 10117 Berlin, Germany; Charité - Universitätsmedizin Berlin, Freie Universität Berlin and Humboldt-Universität zu Berlin, Department of Psychiatry and Neurosciences, Charité Campus Mitte (CCM), Charitéplatz 1, 10117 Berlin, Germany; Charité - Universitätsmedizin Berlin, Freie Universität Berlin and Humboldt-Universität zu Berlin, Department of Psychiatry and Neurosciences, Charité Campus Mitte (CCM), Charitéplatz 1, 10117 Berlin, Germany

**Keywords:** systematic review, noninvasive brain stimulation, cognition, attention, oscillations

## Abstract

Working memory (WM) is essential for reasoning, decision-making, and problem solving. Recently, there has been an increasing effort in improving WM through noninvasive brain stimulation (NIBS), especially transcranial direct and alternating current stimulation (tDCS/tACS). Studies suggest that tDCS and tACS can modulate WM performance, but large variability in research approaches hinders the identification of optimal stimulation protocols and interpretation of study results. Moreover, it is unclear whether tDCS and tACS differentially affect WM. Here, we summarize and compare studies examining the effects of tDCS and tACS on WM performance in healthy adults. Following PRISMA-selection criteria, our systematic review resulted in 43 studies (29 tDCS, 11 tACS, 3 both) with a total of 1826 adult participants. For tDCS, only 4 out of 23 single-session studies reported effects on WM, while 7 out of 9 multi-session experiments showed positive effects on WM training. For tACS, 10 out of 14 studies demonstrated effects on WM, which were frequency dependent and robust for frontoparietal stimulation. Our review revealed no reliable effect of single-session tDCS on WM but moderate effects of multi-session tDCS and single-session tACS. We discuss the implications of these findings and future directions in the emerging research field of NIBS and WM.

## Introduction

In recent years, there has been an increasing effort in improving cognitive functions, including working memory (WM), with noninvasive brain stimulation (NIBS; Begemann et al. 2020). Besides possible benefits especially for people with cognitive deficits, these methods may also provide causal insight into the role of specific neurophysiological measures, such as neural oscillations, for WM functions. Next to transcranial magnetic stimulation (TMS; Pitcher et al. 2021), the most broadly applied NIBS methods are transcranial direct current stimulation (tDCS) and transcranial alternating current stimulation (tACS). Evidence that repetitive TMS of the frontal cortex can improve WM function has been reviewed elsewhere (Brunoni and Vanderhasselt 2014). However, transcranial electric stimulation is more cost effective, easier to apply and has a higher tolerability than TMS (Matsumoto and Ugawa 2017). For these reasons, tDCS/tACS would be easier to integrate into clinical practice.

Depending on the polarity of the electrode situated over the target brain region, tDCS is thought to increase (anodal stimulation) or decrease (cathodal stimulation) cortical excitability by modulating neuronal membrane potential (Stagg and Nitsche 2011). In contrast to tDCS, tACS has been found to modify endogenous neural oscillations in a frequency-specific manner (Herrmann et al. 2013). tACS is thought to operate primarily by resonance (Ali et al. 2013) and entrainment (Helfrich et al. 2014) mechanisms. At the single-cell level, this can be measured in entrainment of spike times (Krause et al. 2019; Johnson et al. 2020). tDCs and tACS may influence WM functions in several ways. Regarding tDCS, cortical excitability and excitation-inhibition balance in the prefrontal cortex has been often related to WM (Polizzotto et al. 2020), whereas for tACS, neuronal synchrony between brain areas such as frontal and parietal cortices, which can be targeted using dual-site montages, is known to be tied to WM performance (Polanía et al. 2012).

Two influential meta-analyses have previously investigated the effects of tDCS on WM (Hill et al. 2016; Mancuso et al. 2016). In their analysis, Hill et al. (2016) reported small offline effects of tDCS on reaction times (RT) in WM tasks, but no effects on accuracy or online effects. However, Mancuso et al. (2016) showed that the weak but significant offline effects of tDCS alone on WM became nonsignificant once the analysis was corrected for publication bias. Nevertheless, the combination of tDCS and WM training exhibited significant effects on performance. Therefore, the authors suggested that tDCS may still have potential for modulating WM function when applied during multi-session WM training. However, this conclusion was based on an integrative analysis of studies with mostly small participant samples. In summary, while there is some evidence that tDCS may improve WM training, there are mixed results regarding the effects of single-session tDCS on WM (Horvath et al. 2015).

In recent years, an increasing number of methodologically advanced studies have been conducted in this research field (Hill et al. 2017; Nikolin, Martin, et al. 2018; Schmicker et al. 2021). Here, we provide an updated overview of studies that have investigated tDCS effects on WM performance. Extending the scope of previous work, we will additionally examine studies that used tACS to target WM performance and will compare the findings of these two methods. Recently, a review of tACS studies targeting memory performance, including WM, has found small to medium effects tACS on WM (Booth et al. 2022). The overarching aim of this review is to provide a comprehensive and integrative overview of studies examining tDCS and tACS effects on WM in healthy adults.

## Methods

### Study inclusion criteria

The following inclusion criteria were applied: (i) studies including healthy adults over 18 years; (ii) randomized controlled studies investigating WM performance using behavioral tests, (iii) application of tDCS and/or tACS regardless of stimulation site, current intensity, stimulation frequency (in tACS), or experimental procedure, and (iv) to diminish the influence of possible spurious effects in small-sample studies (Button et al. 2013), only studies with a minimum of 15 participants in stimulation and the control condition(s) (within-subjects design studies) or control group(s) (between-subjects design studies) were included in this review. This number fits with our literature search, which revealed that the vast majority of studies involved 15 or more participants. Of the 43 studies selected for this review, 16 had sample sizes between 15 and 19. These studies would have been excluded, if we would have applied a more rigorous selection criterion of, e.g., 20 participants. (v) Studies that included a control group or control condition with sham intervention; (vi) Studies that were published or accepted for publication in a peer-reviewed journal and written in English.

### Search strategy

To identify studies, the databases of medical articles MEDLINE (Pubmed) and EMBASE (Ovid) were systematically searched from June 9, 2021, to August 16, 2021. No time restriction was applied. All entries published up until August 2021 were included. Standardized keywords according to the “MeSH” system (Pubmed) and the “Thesaurus” keywords (EMBASE) were used in the search to ensure that all similar and synonymous terms were automatically included. As an example, the search using the “MeSH” Term “transcranial direct current stimulation” includes among others the following keywords: [“tDCS,” “Cathodal Stimulation tDCS,” “Transcranial Alternating Current Stimulation,” “Transcranial Electrical Stimulation,” “Anodal Stimulation Transcranial Direct Current Stimulation.” In this way, all keywords related to tDCS and tACS were considered. Likewise, the keyword WM is subordinated to the MeSH-Term “memory, short term.” Accordingly, the applied search in MEDLINE (Pubmed) was as follows: [“transcranial direct current stimulation”[MeSH Terms] AND “randomized controlled trial”[Publication Type] AND “memory, short term”[MeSH Terms]], and similarly in EMBASE (Ovid): [(Transcranial alternating current stimulation/or Transcranial direct current stimulation/) and working memory/]. The EMBASE search was limited to abstracts and randomized controlled trials. Articles found were screened for relevance by their titles and abstracts. Publications that seemed to meet the inclusion criteria were read in full text. The reference lists of each included article were used to identify other relevant studies that were not previously identified in database searches.

### Extraction of statistical values

In the majority of reviewed studies, the effects of tDCS and/or tACS on WM performance were examined for several experiments (e.g., WM, attention, vigilance). Here, we focus on the main behavioral outcome of the WM task(s). Some studies obtained their behavioral data in the context of neuroimaging experiments, e.g., electroencephalography (EEG) or functional magnetic resonance imaging (fMRI). The statistical values for the neuroimaging findings were not extracted. For the outcome of the primary behavioral analysis and for statistically justified follow-up tests (e.g., follow-up tests when a significant interaction was found in an ANOVA), we extracted statistical values and summarized them in three overview tables. In some articles, follow-up analyses were conducted although they were statistically not justified. For these findings, we highlight that they derive from a secondary data analysis. Moreover, a few articles did not report if they corrected their statistical values for multiple testing. In these cases, we highlight that the values are presumably uncorrected. For one study, we had to recompute the *P*-value based on the *F*-value and the degrees of freedom, because the outcome after outlier removal was reported as “*P* = 0.05” (Ramaraju et al. 2020). The recomputed value was not significant (*P* = 0.052). We evaluated any available performance outcome measure, i.e., accuracy, RT, or *d*’.

**Fig. 1:**
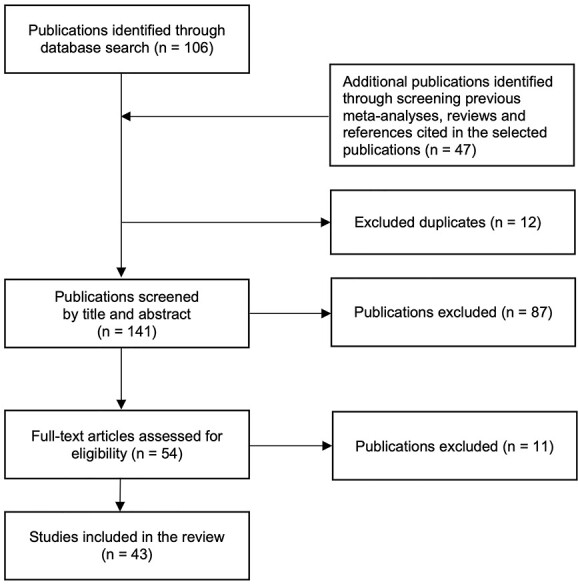
PRISMA chart of study selection.

## Results

### Study selection

The study selection by two authors (DS and RS, with support by DH and SRS) followed the PRISMA-selection criteria (Page et al. 2021). Our systematic database search resulted in 106 studies (Fig. 1). In addition, 47 studies were found by scanning the reference lists for other empirical studies, previous meta-analyses, and systematic reviews. After eliminating 12 duplications, i.e., studies that were found in both databases, a total of 141 publications were taken into consideration. Eighty-seven of these articles were excluded after reading the titles and abstracts. Specifically, research studies were excluded that focused on neurological or psychiatric populations, lacked a sham control group or sham control condition, examined nonhuman primates, or did not perform behavioral tests. The remaining 54 studies were reviewed for eligibility. Out of these, 5 studies were excluded because they did not explicitly assess WM (Meinzer et al. 2015; Murugaraja et al. 2017; Lang et al. 2019; Mansouri et al. 2019; Park et al. 2019). In addition, 6 articles contained only study proposals without final results (Woods et al. 2018; Park et al. 2019; Rajji et al. 2020) or did not include a sham control group (Gonzalez et al. 2018; Emonson et al. 2019). In one study, the sham control group was smaller than 15 (Violante et al. 2017). Taken together, 43 studies met the inclusion criteria for this review.

**Table 1:** Single-session studies on the effects of tDCS on WM.

	Task	Subjects (*N*, age)	Anode	Cathode	Electr. [cm^2^]	Curr. [mA, mA/cm^2^]	Session	Main findings	Statistics^1^
Abellaneda-Pérez et al. (2020)	*n*-back: vis. letters	15 tDCS 14 tACS 15 sham 25.3 y	F3	Ctr supraorb	35	2, 0.057	20-min online and offline	No effect of tDCS on WM performance (*d*’)	n.s.
Berryhill and Jones (2012)	*n*-back: vis. letters and shapes	25 tDCS + sham 63.7 y	F3 & F4	Ctr cheek	35	1.5, 0.043	10-min offline	No main effect of tDCS on CR.^a^ Specific effects on HR in higher educated individuals^b^	^a^n.s. ^b^*F*(1,22) = 5.86 *P* = 0.02, ƞ_p_^2^ = 0.21
Di Rosa et al. (2019)	visuosp. WM	21 tDCS + sham 69.7 y	betw. F3-F7	Ctr shoulder	35	1.5, 0.043	26-min online	No effect of tDCS on RT and CR	n.s. all *P*s > 0.44
Dumont et al. (2021)	visuosp. and vis. letters WM	47 tDCS + sham 24.2 y	P3	Ctr cheek	9	2, 0.22	20-min online	No effect of tDCS on RT and CR	n.s.
Framorando et al. (2021)	*n*-back: vis. letters	17 tDCS + sham 21.8 y	F3	Ctr site F4	25	1, 0.04	11-min offline	No effect of tDCS on RT and CR	n.s. *F*s(1,16) < 0.2 all *P*s > 0.654 ƞ^2^ < 0.012
Fregni et al. (2005)	*n*-back: vis. letters	15 tDCS + sham 20.2 y	F3	Ctr supraorb	35	1, 0.029	10-min online	tDCS improved CR^a^ and reduced ER^b^	^a^ *t*(14) = 3.4 *P* = 0.0042 ^b^*t*(14) = 2.77 *P* = 0.0015
Friehs and Frings (2019)	n-back: vis. letters	42 tDCS 21 sham 24.2 y	F3	left deltoid muscle	9 and 35	0.5, 0.056	20-min online or offline	No diff. Betw. tDCS vs. sham in CR & RT.^2^ Diff. in CR^a^ and RT^b^ betw. Online vs. offline tDCS + sham	^a^ *P* = 0.015 ^b^*P* = 0.029
Gan et al. (2019)	operation span, vis. letters	22 tDCS + sham 21.6 y	P3	P7, Pz, C3, O1	3.14	2, 0.64	20-min online	No effect of tDCS on CR and *d*’	n.s. *P* = 0.079
Hill et al. (2017)	n-back: vis. letters	19 tDCS + HD-tDCS + sham 29.11 y	F3	Fp2; HD-tDCS: Fp1, Fz, C3, F7	12.56; 3.14	1, 0.32	20-min offline	No effect of tDCS and HD-tDCS on RT and *d*’	n.s.
Hill et al. (2018)	*n*-back: vis. letters	16 tDCS + sham 32.81 y	F3	FP1, Fz, C3, F7	3.14	1.5, 0.47	15-min offline	No effect of tDCS on RT and *d*’	n.s.
Hill et al. (2019)	*n*-back: vis. letters	20 tDCS + sham 24.1 y	F3	FP1, Fz, C3, F7	3.14	1.5, 0.48	15-min offline + 15-min online	No effect of online or offline tDCS on RT and *d*’	n.s.
Hoy et al. **(**2013)	*n*-back: vis. letters	17 tDCS + sham 24.7 y	F3	Ctr supraorb	35	1 or 2, 0.029	20-min offline	No effect of tDCS on RT and CR.^a^ Uncorr. effect of 1-mA stim. on 2-back RTs^b^	^a^n.s. ^b^*P* < 0.046
Hoy et al. (2015)	*n*-back: vis. letters	18 tDCS + tACS + sham 29.3 y	F3	Ctr supraorb	35	2, 0.057	20 min. Offline	No effect of tDCS on RT and *d*’	n.s. *P* = 0.605
Karthikeyan et al. (2021)	*n*-back: visuosp.	32 tDCS + sham 26.0 y	F3	Ctr supraorb	35	1, 0.029	10-min online and offline	tDCS improved CR, specificity, and sensitivity online and offline	all *P*s < 0.0022
Maheux-Caron et al. (2021)	*n*-back: vis. letters	34 tDCS + sham 23.8 y	F3	Ctr supraorb	35	2, 0.057	20-min online	No effect of tDCS on CR	n.s. *P* = 0.98
Nikolin et al. (2015)	*n*-back: vis. letters	16 tDCS + sham 21.8 y	F3 & CP5 & P9	4 electr. around stim. site	3.14	2, 0.64	20-min offline	No main effect of tDCS on CR.^a^ Uncorrected effect of DLPFC stim. on RTs^b^	^a^n.s. ^b^*F*(1,15) = 7.51, *P* = 0.02 ƞ^2^ = 0.35
Nikolin, Lauf, et al. (2018)	*n*-back: vis. letters	52 tDCS 26 sham 22.2 y	F3 or P3	4 electr. around stim. site	3.14	2, 0.64	20-min online and offline	No effect of tDCS on RT and CR	n.s. *F*(2,74) = 1.44, *P* = 0.24 ƞ^2^ = 0.04
Nikolin, Martin, et al. (2018) ^3^	*n*-back: vis. letters	40 tDCS 60 sham 22.9 y	F3	F4	16	1 or 2, 0.063	15-min online	No effect of tDCS on RT and CR	n.s. *F*(4,92.2) = 1.0, *P* = 0.414
Ohn et al. (2008)	*n*-back: vis. letters	15 tDCS + sham 26.5 y	F3	Ctr supraorb	25	1, 0.04	30-min offline	No main effect of tDCS on RT, ER, CR.^a^ Uncorr. effect on CR after 30 min^b^	^a^n.s. ^b^*P* < 0.05
Pope et al. (2015)	auditory executive function	42 tDCS 21 shams 21.8 y	F3	Ctr deltoid muscle	25	2, 0.08	20-min offline	tDCS improved CR in a number subtraction task	*F*(2,53) = 11.87 *P* < 0.001
Ramaraju et al. (2020)	*n*-back: letters and shapes	20 tDCS + sham 30.0 y	F3	Ctr supraorb	35	1.5, 0.043	15-min offline	No effects of tDCS on CR^4^	n.s.
Röhner et al. (2018)	*n*-back: vis. letters	30 tDCS + tACS + sham 26.2 y	F3	Ctr shoulder	35	1, 0.029	15-min online and offline	No effect of tDCS on RT and CR	n.s. *F*(2,18) = 0.5 *P* = 0.059
Schmicker et al. (2021)	vis. delayed match-to-sample	38 tDCS 40 sham 23.4 y	F3, F8, Fp2	P3, Pz	35	1.5, 0.043	10-min offline	tDCS differentially affects CR-WM transfer in high vs. low capacity individuals	*F*(1,74) = 7.10 *P* = 0.009, ƞ_p_^2^ = 0.088

^1^Statistical values were derived from the articles and are linked to the main findings via index letters. If available, values are reported for nonsignificant tests.

^2^No significant effect of tDCS vs. sham condition after correction for multiple comparison. Information was provided by the corresponding author.

^3^In this article, 2 tDCS experiments are presented. Exp. 1 included 20 participants per group. Exp. 2 included 20 participants in the tDCS group and 40 participants in 2 sham conditions.

^4^This statistics, i.e., the *P*-value, was recomputed for the outlier corrected *F*-value and DFs reported in the article and was not significant. Betw: Between; CR: Correct responses; Ctr: Contralateral; Curr: Current; Electr: Electrode; ER: Error rate; min: minutes; n.s.: not significant; Stim: Stimulation; Subraorb: Supraorbital; Vis: Visual; Visuosp: Visuospatial.

**Table 2:** Multi-session studies on the effects of tDCS on WM.

	Task	Subjects (*N*, age)	Anode	Cathode	Electr. [cm^2^]	Curr. [mA, mA/cm^2^]	Sessions	Main findings	Statistics^1^
Assecondi et al. (2021)	*n*-back: visuospatial	42 tDCS 42 sham 20.6 y	F4	Ctr supraorb	3.14	2, 0.64	2 × 20 min offline	No main effect of tDCS on CR^a^ but specific effects for individuals with low WM capacity^b^	^a^n.s. ^b^*t*(24) = 6.47, *P* < 0.001
Au et al. (2016)	*n*-back: visuospatial	40 tDCS 22 sham 21.0 y	F3 or F4	Ctr supraorb	35	2, 0.057	7 × 20–25 min offline	tDCS improved CR-WM training performance,^a^ which was preserved for several months^b^	^a^ *P* = 0.002, *d* = 0.77 ^b^*P* = 0.03, *d* = 1.04
Byrne et al. (2020)	vis. backw. recall; *n*-back: letters/digit	16 tDCS 32 sham 23.2 y	F3	Ctr supraorb	25	1, 0.04	3 × 10 min online	No effect of tDCS on CR-WM training	all *P*s > 0.58
Jones et al. (2015)	vis.operation span, *n*-back: visuospatial	54 tDCS 18 sham 64.4 y	F4 or P4	Ctr Cheek	35	1.5, 0.043	10 × 10 min offline	No effect of tDCS on WM training or transfer after 10 sessions.^a^ However, tDCS group performed better in 4-week follow-up^b^	^a^ *F*(1,70) = 0.83, *P* < 0.37 ^b^*F*(1,70) = 7.32, *P* < 0.01 ƞ_p_^2^=0.10
Ke et al. (2019)	*n*-back: vis. letters shapes	15 tDCS 15 sham 22.5 y	F3	Fp1, Fz, C3, FT7	2.5	1.5, 0.6	5 × 25 min online and offline	tDCS improved CR-WM training effect	all *P*s < 0.05
Park et al. (2014)	*n*-back: vis. letters	20 tDCS 20 sham 69.8 y	F3	F4	25	2, 0.08	10 × 30 min. Online	tDCS improved CR-WM training^a^ and RTs^b^.	^a^ *P* = 0.04, ^b^*P* = 0.05
Richmond et al. (2014)	adaptive WM span task: letters/visuospatial	20 tDCS 20 sham 21.0 y	F3	F4	35	1.5, 0.043	10 × 15 min online/offline	tDCS improved WM spans^a^ and enhanced verbal WM^b^	^a^ *F*(1,38) = 4.97 *P* = 0.032 ^b^*F*(1,38) = 27.6 *P* = 0.025
Ruf et al. (2017)	*n*-back: vis. letters and visuospatial	48 tDCS 23 sham 24.4 y	F3 or F4	Ctr deltoid muscle	35	1, 0.029	3 × 20 min online and offline	tDCS improved WM learning curves in trained and untrained exp. domain.	*F*(2,48) = 6.91 *P* < 0.002 ƞ_p_^2^=0.22
Martin et al. **(**2013) ^2^	*n*-back: visuospatial and auditory letters	31 tDCS 21 sham 22.9 y	F4	Ctr deltoid muscle	35	2, 0.057	10 × 30 min online and offline	No online CR-WM training effects when group baseline differences were considered	*F*(1,91,153) = 0.9, *P* = 0.47

^1^Statistical values were derived from the articles and are linked to the main findings via index letters. If available, values are reported for nonsignificant tests.

^2^In this study, one group received tDCS only (*n* = 10) and another group received tDCS plus computer training (*n* = 21). CR: Correct response; CT: Computer training; Stim: Stimulation; Electr: Electrode; Curr: Current; Vis: Visual; Ctr: Contralateral; Subraorb.: Supraorbital; min: minutes; n.s.: not significant.

**Table 3:** Studies on the effects of tACS on WM.

	Task	Subjects (*N*, age)	Electr. config	Electr. [cm^2^]	Curr. [±mA, mA/cm^2^]	Freq. [Hz]	Session	Main findings^*^	Statistics^1^
Abellaneda-Pérez et al. (2020)	*n*-back: vis. letters	14 tACS 15 tDCS 15 sham 25.3	F3-FP2	35	2, 0.057	6	20-min online/offline	No effect of tACS on WM performance (CR, RT, d’)	n.s.
Alekseichuk et al. (2016)	*n*-back: visuospatial	16 tACS + sham 23.5 y	5 electr.:4 electr. 6 cm around AF3	1	6 Hz = 0.6, 0.6 80 Hz = 0.4, 0.4	6 and 6 + 40|60|80| 100|140	10-min online	Theta^a^ and cross-frequency theta–gamma^b^ tACS improved WM performance (*d*’)	^a^ *g* = 0.19, *P* < 0.05 ^b^*g*_*CF-*6,80*p*_ = 0.48, *P* < 0.05
Guo et al. (2021)	color recall	36 tACS + sham 20.9 y	P4-FP2	25	4 Hz: Ø = 1.09, 0.044 7 Hz: Ø = 0.95, 0.038	4 and 7, sep.	20-min online	4 Hz stim. improved,^a^ 7 Hz stim. impaired^b^ capacity of WM in high cap. adults	^a^ *P* = 0.009 ^b^*P* = 0.003
Hoy et al. (2015)	*n*-back: vis. letters	18 tACS + tDCS + sham 29.3 y	F3-Ctr supraorbital area	35	1.5, 0.043	40	20-min offline	No effect of tACS on CR, RT, *d*’	*F*(1,17) = 3.32 *P* = 0.086
Jaušovec et al. (2014)	Corsi block-tapping; digit span; vis. spatial/letter *n*-back	36 tACS + sham 20.4 y	F3 or P3 or P4—above right eyebrow	35	mode = 1.75, 0.05	indiv. theta/alpha, Ø = 5.14	15-min offline	tACS improved aggregated measures of WM span and *n*-back tasks	*F*(1,33) = 21.56 *P* < 0.001 ƞ^2^=.40
Jaušovec and Jaušovec (2014) ^2^	vis. array comparison	24 tACS + sham 20.0 y	F3 or P3 - above right eyebrow	35	mode = 1.75, 0.05	indiv. theta/alpha, Ø = 5.07	15-min offline	tACS over parietal^a^ but not frontal^b^ cortex improved WM capacity	^a^ *t*(11) = 3.94 *P* < 0.002 *d* = 1.14 ^b^*t*(11) = 0.17 *P* < 0.87 *d* = 0.05
Jones et al. (2019)	visuospatial and visual object *n*-back	38 tACS + sham 24.5 y	F4–P4 or F3–F4	25	1, 0.04	4.5	15-min online	Frontoparietal^a^ but not frontal only^b^ tACS improved *d*’ for visual objects	^a^ *t*(37) = 2.33 *P* = 0.03 ^b^*P* > 0.09
Pahor and Jaušovec (2018)	figural/verbal change detection; vis. letters/figures *n*-back	72 tACS + sham 20.4 y	F3-P3 or F4-P4 or P3-P4 or P4-F4	35	Ø = 1.75, 0.05	indiv. theta and gamma, sep.	15-min offline	No effect of tACS on RT and CT	*F*(6,136) = 0.62, *P* = 0.70 ƞ^2^=.03
Polania et al. (2012)	delayed visual letter discr.	18 tACS + sham 26 y	dual-site: F3-Cz and P3-Cz	35	1, 0.029	6 or 35	15-min online	In-phase frontoparietal 6 Hz facilitated RT^a^. Anti-phase tACS prolonged RT^b^	^a^ *t*(17) = 1.76, *P* < 0.05 ^b^*t*(17) = 3.41 *P* < 0.01
Reinhart and Nguyen (2019)	figural change detection	84 tACS, young: 24.5 y, old: 68.8 y	dual-site: 3 electr. left PFC, 3 electr. left TC	1.2	0.6, 0.5 or 1, 0.83 or 1.6, 1.3	indiv. theta or 8	25-min online/offline	In-phase individ. frontotemporal theta tDCS improved CR in elderly	*t*(41) = 3.73 *P* = 0.001 *d* = 0.577
Röhner et al. (2018)	*n*-back: vis. letters	30 tACS + tDCS + sham 26.2 y	F3-P3	35	0.5, 0.014 at each electr.	6 in-phase fronto-parietal.	15-min online/offline	No effect of tACS on RT and CT	n.s.
Sahu and Tseng (2021)	vis. retro-cueing	18 tACS + sham 27 y	F4-P4	25	1.5, 0.06	6	15-min offline	Theta tACS improved CR	*F*(1,17) = 16.39 *P* < 0.001 ƞ_p_^2^=0.491
Thompson et al. (2021)	vis. retro-cueing	51 tACS + sham 24.1 y	P3-P4	35	1.5, 0.043	10 and 35, sep.	20-min online	Parietal gamma tACS improved recall precision	*P* = 0.02, *d* = 0.3
Wolinski et al. (2018)	visuospatial delayed match to sample	32 tACS 25.6	P4-Cz or P4-Ctr supraorbital	35	Ø = 1.23, 0.035	4 and 7, sep.	12-min online	4 Hz stim. improved WM capacity.^a^ 7 Hz stim. reduced WM capacity^b^	^a^ *t*(15) = 2.28 *P* = 0.019 *d* = 0.57 ^b^*t*(15) = −1.78 *P* = 0.0047 *d* = 0.44

^1^Statistical values were derived from the empirical articles and are linked to the main findings via index letters. If available, values are reported for nonsignificant tests.

^2^In this study, a subsample of Jaušovec and Jaušovec (2014) was investigated. Stim: Stimulation; Electr: Electrode; Vis: Visual; CR: Correct responses; Ctr: Contralateral; PFC: Prefrontal cortex; TC: Temporal cortex; Indiv.: Individual; min: minutes; sep: separately; n.s.: not significant.

The sample sizes of the 43 studies varied from a predefined minimum of 15 (Fregni et al. 2005) to a maximum of 100 (Nikolin, Martin, et al. 2018), with a total of 1826 participants. Thirty-eight studies involved a young healthy cohort (mean age across studies: 23.6) and 5 studies examined older individuals (mean age across studies: 67; Berryhill and Jones 2012; Di Rosa et al. 2019; Jones et al. 2015; Park et al. 2014; Reinhart and Nguyen 2019). All studies report high tolerability for tACS as well as tDCS. Adverse events mentioned were mild itching, tingling, headache, or local skin redness (Fregni et al. 2005; Berryhill and Jones 2012; Jaušovec et al. 2014; Au et al. 2016; Nikolin, Martin, et al. 2018; Reinhart and Nguyen 2019; Ramaraju et al. 2020; Stonsaovapak et al. 2020). The studies’ outlines, methodology, and main findings are provided in Tables 1–3. 

### Studies examining tDCS effects on WM

In total, 32 tDCS studies met our selection criteria. A previous meta-analysis has indicated that tDCS may primarily affect WM training (Mancuso et al. 2016). Therefore, we separately review studies employing single-session (*n* = 23) and multi-session (*n* = 9) tDCS experiments. We will first describe the general effect pattern across all studies, independent of differences in experimental paradigms, stimulation intensities, or locations of the stimulation electrodes. Then, we will inspect whether any effects may be related to specific stimulation parameters, experimental conditions, or other factors such as sample size. With the exception of 2 single-session tDCS experiments, all studies included the stimulation of the frontal cortex, i.e., placement of the anode at F3 or F4.

For single-session tDCS experiments, 4 out of 23 studies, i.e., 17%, revealed general (Table 1; Fregni et al. 2005; Pope et al. 2015; Karthikeyan et al. 2021) or subgroup-specific (Schmicker et al. 2021) main effects on WM performance. Interestingly, 3 studies that reported effects involved attentionally demanding tasks (Fregni et al. 2005; Pope et al. 2015; Karthikeyan et al. 2021). Fregni et al. (2005) investigated online effects of tDCS over the left dorsolateral prefrontal cortex (DLPFC) on performance in a 3-back letter task. The study showed small improvements in hit rates and reductions in error rates for the tDCS compared with the sham control condition. In another study, Karthikeyan et al. (2021) examined effects of tDCS over the DLPFC in a fatiguing visuospatial *n*-back task. The fatigue-related decline in WM performance was smaller in the tDCS compared with the sham group. Pope et al. (2015) investigated the effects of anodal and cathodal tDCS over the DLPFC and sham stimulation on a paced auditory serial number addition and subtraction task. The authors observed improved response accuracies for anodal tDCS in the attentionally demanding subtraction task, but not in the addition task. In contrast to these findings, several other studies that also included demanding tasks did not report effects of tDCS on WM (Hoy et al. 2015; Nikolin, Lauf, et al. 2018; Nikolin, Martin, et al. 2018; Abellaneda-Pérez et al. 2020; Maheux-Caron et al. 2021). One study, which examined offline and online effects of tDCS in different groups, showed an improved WM performance in a 3-back task for offline vs. combined online and sham stimulation (Friehs and Frings 2019). Moreover, there were differences in online and offline tDCS effects on WM, indicating a role of stimulation timing. However, the effects on WM performance did not significantly differ between the tDCS groups and the sham control group. Hence, there are mixed findings regarding possible effects of tDCS on performance in attentionally demanding WM tasks. More recently, Schmicker et al. (2021) observed improved training-induced WM transfer in participants with a high WM-capacity, but not in participants with a low WM-capacity. Similarly, an exploratory post-hoc analysis by Berryhill and Jones (2012) showed subgroup-specific tDCS effects on WM performance in a small subsample of highly educated participants, which was not found in participants with a low educational level. Further research is required to address whether single-session tDCS can improve WM performance, especially in demanding tasks, and whether single-session tDCS may differentially influence performance in individuals with high vs. low WM capacities. For the 4 single-session tDCS studies that revealed significant effects on WM performance compared with the 19 studies that did not reveal effects, we did not observe systematic differences in terms of sample sizes or the locations or intensities of the stimulation. Thus, there were no specific stimulation parameters that predicted the effects obtained in the 4 studies with significant findings (Fregni et al. 2005; Pope et al. 2015; Karthikeyan et al. 2021; Schmicker et al. 2021).

**Fig. 2:**
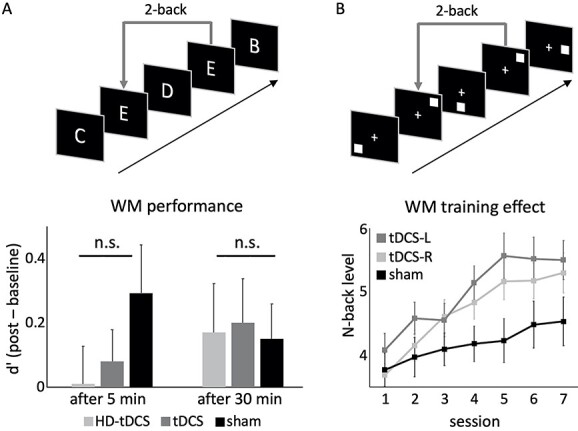
Effects of single-session and multi-session tDCS on WM. A) A single-session tDCS experiment revealed no differences between frontal high-density tDCS (HD-tDCS), regular tDCS, and sham after 5 or 30 min on performance in a verbal 2-back task. Redrawn, with the authors permission after Hill et al. (2017). B) Over the course of 7 training sessions, tDCS over the left (tDCS-L) or right (tDCS-R) DLPFC improves performance in a visuospatial 2-back task. Redrawn, with the authors permission after Au et al. (2016).

The vast majority, i.e., 83%, of single-session tDCS studies did not reveal online (Nikolin, Lauf, et al. 2018; Nikolin, Martin, et al. 2018; Röhner et al. 2018; Di Rosa et al. 2019; Abellaneda-Pérez et al. 2020; Dumont et al. 2021; Maheux-Caron et al. 2021) and/or offline effects (Ohn et al. 2008; Berryhill and Jones 2012; Hoy et al. 2013, 2015; Nikolin et al. 2015; Hill et al. 2017, 2018, 2019; Nikolin, Lauf, et al. 2018; Röhner et al. 2018; Abellaneda-Pérez et al. 2020; Ramaraju et al. 2020; Framorando et al. 2021). For example, Hill et al. (2017) did not find offline effects of tDCS or high-density tDCS over frontal cortex on performance in a verbal *n*-back task (Fig. 2A). Taken together, the reviewed studies suggest that single-session tDCS has no reliable influence on WM performance.

For multi-session tDCS experiments (Table 2), 7 out of 9 studies, i.e., 78%, revealed tDCS-related significant general improvements (Park et al. 2014; Richmond et al. 2014; Jones et al. 2015; Au et al. 2016; Ruf et al. 2017; Ke et al. 2019) or subgroup-specific improvements (Assecondi et al. 2021) of WM training. Au et al. (2016) investigated the effects of tDCS over the DLPFC on visuospatial WM training and found that stimulation improved WM over the course of 7 sessions (Fig. 2B). This improvement was maintained over several months. In another study, Jones et al. (2015) examined the training effects of 10 sessions of tDCS delivered over the DLPFC or the parietal cortex on a visuospatial *n*-back and a visual operation span task. After the 10 sessions, no effects were found. However, the tDCS compared with the sham group showed better WM performance at a 1-month follow-up. Effects of tDCS over the DLPFC on performances in *n*-back tasks with letters and shapes have been examined across 5 sessions by Ke et al. (2019). The study revealed higher learning rates in the tDCS group compared with the control group.

Previously, a cross-over double-blinded study by Park et al. (2014) that included 10 training sessions of a visual *n*-back task showed improved training effects in the tDCS group. Moreover, Richmond et al. (2014) investigated WM training effects on adaptive WM span tasks with letters and visuospatial stimuli. They observed improved training effects on WM span and verbal WM, when tDCS over the DLPFC was applied during 10 sessions. In another study, Ruf et al. (2017) showed that tDCS over the DLPFC can improve learning curves in visual *n*-back tasks for trained and untrained experimental domains across three sessions. Notably, these effects were still detectable after 9 months. However, 2 multi-session tDCS studies, which involved only a few sessions, did not find effects of DLPFC stimulation on WM (Byrne et al. 2020; Assecondi et al. 2021). Assecondi et al. (2021) examined tDCS effects on a visuospatial *n*-back task across 2 sessions. While the authors did not find any effects of tDCS on WM training on a group level, they did observe that individuals with low WM capacity benefited from the combination of tDCS and strategy instructions. Byrne et al. (2020) investigated tDCS effects on visual *n*-back tasks during 3 training sessions but did not find stimulation effects on WM. Hence, it could be that a larger number of training sessions are required to induce reliable tDCS effects on WM (but see, Ruf et al. 2017). Such a notion is supported by an earlier multi-session study comprising 10 training sessions of visual and auditory *n*-back tasks that showed a significant training effect of tDCS (Martin et al. 2013). However, this effect becomes nonsignificant when baseline differences between study groups are included in the analysis. Collectively, the reviewed studies show that multi-session tDCS has moderate positive effects (ranging from small to large) on WM training. In summary, the majority of multi-session tDCS studies revealed positive effects of stimulation on WM training. These effects seem to be more reliable when the studies included a higher number of training sessions and there is some evidence that they are maintained for weeks to months after the WM training.

### Studies examining tACS effects on WM

The literature review identified 14 tACS studies that met the selection criteria (Table 3). All experiments investigated the effects of tACS on WM in a single session. Compared with tDCS studies, there was a larger variability of stimulation approaches across tACS studies, especially due to differences in stimulation frequencies. We will first describe the general effect pattern across all studies, independent of differences in stimulation parameters and paradigms, and will then inspect whether any effects may relate to specific stimulation parameters or experimental conditions.

Ten out of 14 studies, i.e., 71%, revealed significant effects of tACS on WM performance (Polanía et al. 2012; Jaušovec et al. 2014; Jaušovec and Jaušovec 2014; Alekseichuk et al. 2016; Wolinski et al. 2018; Jones et al. 2019; Reinhart and Nguyen 2019; Guo et al. 2021; Sahu and Tseng 2021; Thompson et al. 2021). Most studies focused on the effects of frontal and/or parietal tACS tuned to either 1 frequency or 2 frequencies. For instance, Jaušovec et al. (2014) observed positive effects of individual theta/alpha tACS on aggregated WM performance across different paradigms when delivered over the frontal or parietal cortex. Effects were stronger for parietal compared with frontal tACS.

Examining a subsample of participants from this study, Jaušovec and Jaušovec (2014) found an improved performance in a visual array comparison task for parietal but not for frontal individual theta/alpha tACS. Evidence for a superiority of frontoparietal compared with frontal-only theta (4.5 Hz) tACS has been obtained by Jones et al. (2019). The authors observed improved performance in visual object *n*-back tasks for frontoparietal but not for frontal-only stimulation. More recently, Thompson et al. (2021) examined the effects of alpha (10 Hz) and high beta/low gamma (35 Hz) parietal tACS. The study evidenced improved WM performance in a visual retro-cuing task for gamma stimulation but not for alpha stimulation.

Another study by Wolinski et al. (2018) compared the effects of theta (4 and 7 Hz) tACS over the parietal cortex on WM capacity. The authors found that 4-Hz tACS improved WM capacity, whereas 7-Hz stimulation reduced it. Similarly, Guo et al. (2021) observed a deteriorating influence of 7-Hz tACS over frontoparietal areas on WM, specifically in participants with a high WM capacity. Somewhat contradicting these findings, Sahu and Tseng (2021) observed an improved WM performance in a visual retro-cueing paradigm for theta (6 Hz) tACS over frontoparietal areas. Two further studies examined the effects of theta (6 Hz; Abellaneda-Pérez et al. 2020) or gamma (40 Hz; Hoy et al. 2015) tACS over the frontal cortex on verbal *n*-back task performance. Neither study revealed effects of tACS.

Finally, an experiment comprising 4 different stimulation site groups, 4 experimental conditions, 2 stimulation conditions (tACS vs. sham), and 2 stimulation frequencies (individual theta vs. gamma) did not yield any effects of tACS on WM performance (Pahor and Jaušovec 2018). This multifactorial experiment included only 18 participants in each stimulation-site group, and hence, it presumably lacked necessary statistical power to detect small or moderate effects. Taken together, there are mixed findings when tACS is applied to a specific brain region and at a specific frequency: the stimulation can improve or deteriorate WM performance. Nevertheless, most studies found moderate effects of single frequency tACS on WM performance. There is some evidence that 2 preconditions could be vital for this effect: stimulation is in the theta band and localized to the parietal cortex, either alone or jointly with the frontal cortex. A similar conclusion has recently been drawn in a review by Booth et al. (2022), who empathized that stimulation effects on WM were particularly robust for posterior theta stimulation.

In addition to these experiments, 3 methodologically advanced studies have further improved our understanding of how tACS affects WM performance (Polanía et al. 2012; Alekseichuk et al. 2016; Reinhart and Nguyen 2019). In a dual-site stimulation study, Polanía et al. (2012) applied theta (6 Hz) or high beta/low gamma (35 Hz) tACS simultaneously over frontal and parietal areas during a delayed visual letter discrimination task. Across stimulation sites, tACS was applied with a relative 0° (i.e., in-phase) or 180° phase (i.e., anti-phase) difference. For in-phase dual-site theta stimulation, the authors observed RT facilitation effects, whereas for anti-phase theta stimulation, they found prolonged RTs compared with sham. In contrast, 35-Hz stimulation did not affect behavioral performance.

**Fig. 3:**
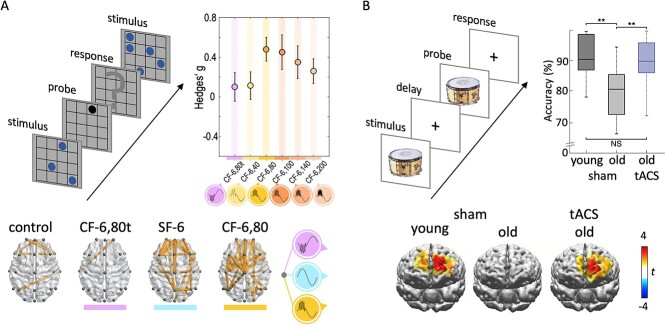
Effects of tACS on WM performance and neural synchrony. A) Participants in this experiment performed a visuospatial match-to-sample test (upper left). Cross-frequency theta (6 Hz) and gamma (40, 80, 100, 140, 200) simulation improved WM performance compared with sham (upper right). The effects were smaller when 80-Hz bursts were applied at the trough of 6 Hz (CF-6,80 t) compared when they were presented at the peak of 6 Hz (CF-6,80). Off-line recorded resting state EEG (lower panel) revealed increased phase connectivity after the 6 Hz only (SF-6) stimulation and after the 80-Hz stimulation applied at peaks of 6-Hz stimulation. Adapted from Alekseichuk et al. (2016), with permission from Elsevier. B) Participants in this experiment performed an object match-to-sample test (upper left). Effects of dual-site frontotemporal tACS were examined in a group of young and old adults. In the sham condition, old adults performed worse than young adults (upper right). In phase-frontotemporal individual, theta stimulation improved WM performance in old adults. This behavioral effect was paralleled by a restoration of offline recorded EEG theta-band phase-locking between the frontal and the temporal cortex in old adults following tACS (lower panel). Adapted from Reinhart and Nguyen (2019) with permission from Springer Nature.

In another study, Alekseichuk et al. (2016) investigated the effects of theta (6 Hz) and cross-frequency theta (6 Hz) and gamma (40, 80, 100, 140, 200 Hz) tACS over the frontal cortex on performance in a visual *n*-back task (Fig. 3A). The study showed improved WM performance for theta stimulation compared with sham. Interestingly, the cross-frequency 6 + 80 Hz stimulation improved performance only when 80-Hz bursts were applied at the peak of 6-Hz tACS, but not when they were applied at the trough of 6-Hz tACS. In addition, the analysis of off-line recorded resting-state EEG data showed an increased phase connectivity for the combined 6- and 80-Hz stimulation, when applied at the peak of the 6-Hz wave, but not for the 80-Hz stimulation applied at troughs of 6-Hz stimulation. Recently, a WM-enhancing offline effect of combined 6- and 80-Hz stimulation was also observed in a combined tACS-TMS study (Hosseinian et al. 2021). In this study, performance in an *n*-back task was improved for 6-Hz tACS-TMS alone.

In a seminal study comprising 4 experiments, Reinhart and Nguyen (2019) examined the effects of dual-site theta tACS over frontal and temporal cortex on performance in a visual object match-to-sample task (Fig. 3B). Effects of various tACS protocols (e.g., individual theta or fixed 8-Hz stimulation; stimulation of one or both sites; in-phase or anti-phase dual-site stimulation) on WM were investigated and compared between larger (*n* > 40) cohorts of younger and older adults. In addition, offline effects of tACS on theta-gamma phase-amplitude coupling at frontal and temporal sites, as well as theta phase synchronization between frontal and temporal sites, were investigated. At the behavioral level, the authors observed a rapid improvement of WM performance for in-phase frontotemporal individual theta stimulation specifically in older adults. These effects outlasted 50 min poststimulation. There was also some evidence for positive tACS effects on WM performance in a smaller (*n* = 14) group of young adults with poorer WM performance. This suggests that the dual-site frontotemporal stimulation in the study is particularly effective in adults with reduced WM performance, such as the elderly. In older adults, the behavioral effects of tACS were paralleled by an offline observed increase in memory-related theta–gamma phase-amplitude coupling in the temporal lobe as well as an increased theta phase synchronization between temporal and frontal cortex. Hence, this study provides strong evidence that dual-site tACS can effectively improve WM function, especially in the elderly. In summary, there is ample evidence that tACS can have rapid effects on WM performance and WM-related neural processing, particularly neural oscillations. The direction and magnitude of effects seem to depend on the stimulation site(s) and stimulation frequency protocols. For a more explicit description of the complex effects of tACS on WM and long-term memory, see Booth et al. (2022).

Lastly, 4 studies did not report effects of tACS on WM performance (Hoy et al. 2015; Pahor and Jaušovec 2018; Röhner et al. 2018; Abellaneda-Pérez et al. 2020). In 2 of these studies, tACS was applied over frontal cortex only (Hoy et al. 2015; Abellaneda-Pérez et al. 2020). This indicates that tACS over the frontal cortex alone may not reliably affect WM performance (Jaušovec and Jaušovec 2014; Jones et al. 2019; but see Alekseichuk et al. 2016). No other systematic differences in terms of sample size and the location or intensity of the stimulation were found between studies that did or did not report effects of tACS on WM.

### Studies comparing effects of tDCS and tACS on WM

Three studies were identified in which the effects of single-session tDCS and tACS on WM were directly compared (Hoy et al. 2015; Röhner et al. 2018; Abellaneda-Pérez et al. 2020). Two of these studies examined the effects of tDCS and theta (6 Hz; Abellaneda-Pérez et al. 2020) or gamma (40 Hz; Hoy et al. 2015) tACS delivered over the frontal cortex on performance in a verbal *n*-back task. Neither study found effects on WM performance, which is in line with other studies that have failed to show behavioral effects of tDCS (e.g., Hill et al. 2017; Nikolin, Lauf, et al. 2018; Nikolin, Martin, et al. 2018) or single frequency tACS (Jaušovec and Jaušovec 2014; Jones et al. 2019) delivered over the frontal cortex. Nevertheless, in the study by Abellaneda-Pérez et al. (2020), the comparison of offline vs. online acquired fMRI data during an *n*-back task revealed differential blood oxygen level-dependent (BOLD) responses and functional connectivity between tDCS and tACS in brain areas of the default mode network. While not behaviorally relevant, these findings indicate differential online and offline effects of tACS and tDCS during WM processing. Finally, Röhner et al. (2018) observed an enhanced RT facilitation effect in an *n*-back task for theta (6 Hz) tACS over frontoparietal areas compared with tDCS. However, this effect was found in a subgroup that only consisted of 10 participants. Therefore, this finding should be interpreted as preliminary evidence that single-session tACS may be superior to single-session tDCS when the effects on WM are investigated in the same individuals. Taken together, there are only a few studies that have directly compared the effects of tACS and tDCS on WM. Given the heterogeneity of research approaches and findings across studies, no summarizing conclusions can be drawn.

## Discussion

In this review, we summarized and integrated the findings of 43 PRISMA-selected studies focusing on the effects of tACS and/or tDCS on WM. The review revealed 3 main findings: (i) single-session tDCS experiments show no reliable effects on WM performance. (ii) multi-session tDCS can have moderate positive effects on WM training. (iii) Depending on the stimulation protocol, single-session tACS can either cause a moderate deterioration or an improvement of WM performance. No multi-session study concerning tACS effects on WM was available.

### Effects of tDCS on WM

The absence of reliable single-session tDCS effects on WM performance is in line with the previous meta-analysis by Mancuso et al. (2016), which showed that the small poststimulation effects of tDCS on WM, as reported by Hill et al. (2016), become nonsignificant if the analysis was corrected for publication bias. Sixteen out of the 23 single-session tDCS experiments included in this review were published in the last 5 years, i.e., 2017–2021. Only 2 of them showed effects of tDCS on WM (Karthikeyan et al. 2021; Schmicker et al. 2021). While the majority of reviewed studies did not report effects, there is some evidence that tDCS may be capable of influencing WM performance when the task is highly demanding (Fregni et al. 2005; Karthikeyan et al. 2021). Moreover, it could be that the influence of tDCS on WM may be stronger for online than for offline monitored WM performance (Friehs and Frings 2019). Finally, tDCS effects on WM may be specific to individuals with higher WM capacity (Schmicker et al. 2021). Further studies are required to test these assumptions. In summary, our updated review reinforces the conclusion that single-session tDCS has no reliable effect on WM.

Compared with single-session tDCS, a different picture of findings emerged for multi-session experiments. In their review, Mancuso et al. (2016) had already hypothesized that the potential of tDCS lies in its use during WM training. This conclusion was drawn from 10 studies, but only 3 of them included larger participant samples (*n* ≥ 15; Martin et al. 2013; Park et al. 2014; Richmond et al. 2014). These 3 experiments were also included in the current review, together with 6 more recent experiments (Jones et al. 2015; Au et al. 2016; Ruf et al. 2017; Ke et al. 2019; Byrne et al. 2020; Assecondi et al. 2021). Four of the more recent multi-session tDCS experiments reported the improvements of WM-training in their primary analysis (Jones et al. 2015; Au et al. 2016; Ruf et al. 2017; Ke et al. 2019) and 1 experiment showed effects in a secondary analysis (Assecondi et al. 2021). The only recent study that did not show effects included only 3 training sessions (Byrne et al. 2020). Thus, it is possible that a larger number of training sessions is required in order to obtain detectable and lasting tDCS effects on WM training. The dependence of WM training effects on the number of tDCS sessions remains to be elucidated.

An interesting finding reported by some studies was that the effects of multi-session tDCS on WM training can be detectable for weeks to months after the sessions (Jones et al. 2015; Au et al. 2016; Ruf et al. 2017). Due to this characteristic, multi-session tDCS could potentially become a tool to improve WM and other cognitive functions also in clinical populations such as neuropsychiatric disorders, e.g., mood or schizophrenia-spectrum disorders (Ciullo et al. 2021) or patients with Alzheimer’s disease or mild cognitive impairments (Inagawa et al. 2019). In summary, our review supports the notion that single-session tDCS has no reliable effects on WM performance, whereas multi-session tDCS can have moderate positive effects on WM training.

### Effects of tACS on WM

The tACS studies included in this review revealed a moderate effect on WM performance, ranging from small to large effects within each of these studies. Two dual-site stimulation studies targeting frontoparietal (Polanía et al. 2012) and frontotemporal (Reinhart and Nguyen 2019) brain regions showed a phase-dependency of theta tACS effects on WM. The performance in both studies was enhanced for in-phase theta-stimulation across stimulation-sites, whereas it deteriorated for anti-phase stimulation. Dual-site theta tACS further contributed to an increased memory-related theta-gamma phase-amplitude coupling and theta phase synchronization between the temporal and frontal cortex, especially in the elderly (Reinhart and Nguyen 2019). Another study used a combined theta–gamma tACS protocol and showed improvements in WM performance specifically when gamma-burst stimuli are presented at the peak but not at the trough of the theta phase (Alekseichuk et al. 2016). There is ample evidence from human (Fell and Axmacher 2011; Rajji et al. 2017; Berger et al. 2019) and animal (Siegel et al. 2009; Fujisawa and Buzsáki 2011; Lisman and Jensen 2013) research that cross-frequency theta–gamma phase-amplitude coupling and theta phase synchronization are neural mechanisms underlying or supporting WM processes. From this perspective, the available findings suggest that tACS can interact with endogenous synchronization mechanisms during WM, in particular when the stimulation protocol resembles the intrinsic oscillatory properties of neural networks (Alekseichuk et al. 2019).

This observation from WM studies is in line with the proposal that the effect of tACS on brain activity and behavior is frequency-specific because they interact with endogenous neural oscillations at the target frequency (Herrmann et al. 2013; Rach et al. 2014). Hence, compared with tDCS, where effects mainly depend on the electrode position and current intensity, the stimulation frequency adds another dimension, which likely contributes to the large variability of tACS effects on WM. For instance, Reinhart and Nguyen (2019) found that a necessary condition for improving WM via tACS was adaptation of the stimulation frequency to each participant’s individual peak frequency. This is in line with other studies showing that the mismatch between a fixed tACS frequency and variable individual peak frequency contributes to interindividual variability in stimulation outcomes (Ali et al. 2013; Herrmann et al. 2013). In contrast to the positive influence of multi-session tDCS, the effects of tACS on performance are found for both directions: improvement and deterioration (e.g., Chander et al. 2016; Wolinski et al. 2018; Guo et al. 2021). This suggests that the externally applied tACS can either facilitate or interfere with memory-related neural synchrony in the brain.

Of particular, interest was that several experiments showed reliable effects of frontoparietal theta tACS on WM performance (Polanía et al. 2012; Jones et al. 2019; Guo et al. 2021; Sahu and Tseng 2021). Electrophysiological studies in healthy individuals (Berger et al. 2019; Jones et al. 2020) and in patients with prefrontal cortex lesions (Johnson et al. 2017) have found a frontoparietal coupling of theta and gamma oscillations during WM. As shown by Alekseichuk et al. (2016), synchronous dual-site theta tACS over frontal and parietal cortex during a WM-task was associated with an increase in offline recorded global phase connectivity during rest. Hence, synchronous stimulation of fronto-parietal networks during WM tasks can presumably alter memory-related network processing, as expressing in neural synchrony.

In summary, the reviewed studies demonstrate that tACS has differential effects on WM. In addition to the electrode locations and the intensity of the current, the frequency stimulation protocol, e.g., individual adapted frequency vs. fixed frequency; single frequency stimulation vs. cross-frequency stimulation; phase-relationships across different stimulation sites; and cross-frequency phase-burst relationships, seems to determine the influence of tACS on WM. Future studies in the field should take this into account when preparing the tACS frequency protocol and also consider findings on the intrinsic oscillatory properties of WM. In this regard, recent advances in the development of closed-loop tACS may provide a promising new avenue that enables the dynamic real-time adaptation of the electrical stimulation to endogenous neural oscillations (Haslacher et al. 2021).

### Differential effects of tDCS and tACS on neural processes

The observed differential effects between tDCS and tACS on WM performance likely relate to differences in their influence on neural processes. In tDCS, a weak electric current is applied to the scalp, traveling in a unipolar direction between 2 surface electrodes. Such noninvasive stimulation is thought to alter neural firing thresholds by depolarizing or hyperpolarizing resting membrane potentials of neurons (Nitsche et al. 2008; Stagg and Nitsche 2011). While anodal stimulation was found to increase cortical excitability, cathodal stimulation decreased excitability (Nitsche and Paulus 2000). Along with effects on the neural membrane potential, tDCS is known to influence neurotransmitter concentrations in the target area, particularly by reducing gamma-aminobutyric acid (GABA) during anodal stimulation, leading to increased excitability (Stagg et al. 2011). While electric field strengths associated with tDCS may not alter firing rates in the targeted area, it was shown that tDCS can enhance low-frequency (1–15 Hz) brain oscillations and increase coherence between distant brain areas influencing behavior (Krause et al. 2017). During tACS, a weak current alternates periodically between at least 2 electrodes at a specific frequency. The often reported aftereffects of tACS on EEG connectivity may relate to the modulation of spike-timing-dependent plasticity (Schwab et al. 2021) and there is ample evidence that tACS modifies endogenous neural oscillations in a frequency-specific manner (Ali et al. 2013; Herrmann et al. 2013). Moreover, it is thought that tACS interacts with ongoing oscillations by entraining neural spike timing (Krause et al. 2019; Jones et al. 2020). Neural oscillations, which are a putative target of NIBS, play an important role in WM (Sauseng et al. 2010; Schneider et al. 2011; Roux and Uhlhaas 2014; Miller et al. 2018; Michail et al. 2021). While tDCS may not modulate neural oscillations in a frequency-specific manner, a smaller sample multi-session study, in which tDCS was applied over parietal and frontal cortex, i.e., alternating across sessions, revealed that the positive effects of stimulation on WM performance were paralleled by modulations in frontoparietal theta oscillations and gamma activity (Jones et al. 2020). This indicates that multi-session tDCS, comparable to tACS, can influence memory-related neural synchrony.

### Limitations

This review has some limitations. It is possible that the search strategy missed relevant studies. MeSH and Thesaurus terms were used to identify keywords and any variations thereof to ensure all available articles were found, even if deviating terminology was used. Yet, it is possible that other terms would have identified additional or other results. To ensure that we detected all studies that fit our selection criteria, we thoroughly scanned the reference lists of the preselected empirical articles, previous meta-analyses, and systematic reviews. Second, the literature search was restricted to peer-reviewed randomized controlled trials written in English. This excluded articles that were unpublished or published elsewhere, e.g., on a preprint platform without peer-review. Therefore, a publication bias cannot be ruled out. Third, only a limited number of multi-session studies (*n* = 9) fit our selection criteria. Although the majority of these studies revealed significant effects, additional studies with a higher number of participants are required to further test our conclusion that multi-session tDCS can moderately improve WM-training. Ideally, these studies would have longitudinal setups. Finally, there is a substantial heterogeneity of experimental paradigms, stimulation protocols, and study populations across the reviewed studies. Moreover, in most experiments, the effects of tDCS/tACS were reported on different WM paradigms, and often these statistical analyses were not sufficiently corrected for multiple testing. In addition, in many cases, other statistical values than those we selected appeared also relevant. Hence, a meta-analysis for the extracted values would have required a much narrower research focus than our review, e.g., examining only one specific paradigm and/or stimulation protocol. Therefore, we did not conduct a meta-analysis for the selected studies and decided instead to present the findings in the framework of a comprehensive literature review.

## Conclusion

We have provided an integrative overview on 43 tDCS/tACS studies investigating the effects on WM performance. Our review revealed that the two methods differentially affect WM. Consolidating the conclusion of a previous meta-analysis (Mancuso et al. 2016), our review suggests that single-session tDCS has no reliable effects on WM. However, there is evidence that multi-session tDCS improves WM performance and that these effects are maintained for weeks to months after the training. This suggests that the repeated application of tDCS can improve neural plasticity during WM training, consistent with results linking repeated tDCS to glutamate-mediated plasticity (Stagg and Nitsche 2011). A different picture of findings emerged for tACS. Given the large variability in protocols that can arise out of the crucial choices of stimulation frequency, electrode position, and phase relationships, the effects of tACS were found in both directions: improvement or deterioration of performance. This implies that tACS can causally and rapidly influence WM processing. The heterogeneity in findings presumably derives from complex modulation patterns of tACS on WM-related neural oscillations. Our review establishes tACS and multi-session tDCS as noninvasive stimulation methods that can improve WM performance. Hence, tDCS and tACS could potentially become a tool to enhance WM and other cognitive functions in clinical populations, such as patients diagnosed with neurodegenerative or neuropsychiatric disorders (Mondino et al. 2014; Reinhart and Nguyen 2019).
